# Misdiagnoses of transthyretin amyloidosis: a clinical and electrodiagnostic study

**DOI:** 10.1186/1750-1172-10-S1-O13

**Published:** 2015-11-02

**Authors:** Andrea Cortese, Alessandro Lozza, Elisa Vegezzi, Enrico Alfonsi, Arrigo Moglia, Giampaolo Merlini, Laura Obici

**Affiliations:** 1 C. Mondino National Neurological Institute, Department of Neurological Sciences, 27100, Pavia, Italy; 2 Amyloidosis Research and Treatment Center, Department of Molecular Medicine, Fondazione Istituto Di Ricovero e Cura a Carattere Scientifico Policlinico San Matteo and University of Pavia, 27100, Pavia, Italy

## Background

Misdiagnosis of ATTR and late diagnosis may be detrimental hampering adequate management and delaying therapy onset. Objective of the present study was to investigate in a large single-centre cohort of genetically–confirmed ATTR patients the prevalence, type and causes of misdiagnoses. Given the high frequency of cases erroneously diagnosed as having chronic inflammatory demyelinating polyneuropathy (CIDP), we investigated the electrodiagnostic (EDx) features which can help distinguish ATTR from CIDP.

## Methods

Retrospective study design. Review of clinical notes and EDx studies of ATTR patients referred to Amyloid Research and Treatment Centre and C. Mondino National Neurological Institute (Pavia) between 1999 and 2013. EDx of thirty-five patients diagnosed with CIDP were used as control for comparison.

## Results

Out of 150 patients with ATTR 51(32%) were initially misdiagnosed including 30(59%) CIDP and 11(22%) lumbar spinal stenosis. Eleven (22%) patients underwent spine surgery and 38(74%) were treated with immunotherapies. Patients misdiagnosed had a significant longer delay before diagnosis of 47±3.7 months vs 34±2.7 months (p= 0.01). Lack of family history and onset after 56 years were significantly associated with misdiagnosis(p<0.01). Out of 30 patients misdiagnosed as having CIDP, 17 had original EDx available for review. Six (35%) had definite and 3(17%) possible CIDP according to EFNS criteria, while 8(47%) did not show demyelinating features. Eleven(37%) had a negative tissue biopsy and 4/5(80%) had raised proteins in cerebrospinal fluid (CSF). We next compared EDx of 53 ATTR with EDx from 35 matched CIDP patients. Conduction slowing and prolongation of distal motor latencies were less prominent in ATTR vs CIDP while conduction blocs were almost invariably absent in ATTR. Conversely, in ATTR motor nerves were more often not excitable both at upper and lower limbs.

## Conclusions

ATTR was misdiagnosed in 1/3 of cases, particularly in patients with late onset and without family history. CIDP was the most common alternative diagnosis, which was supported by EFNS EDx criteria for demyelinating neuropathy in half of them. However conduction slowing is less prominent in ATTR while severe axonal loss is the major EDx feature. DNA testing for TTR should be performed in patients with progressive axonal or mixed axonal-demyelinating peripheral neuropathy, who do not respond to immunotherapies, regardless the lack of family history and a late onset. Raised proteins in CSF and a negative biopsy do not rule out the diagnosis of ATTR.

